# Health-related quality of life and function in middle-aged individuals with thalidomide embryopathy

**DOI:** 10.1007/s11832-016-0797-6

**Published:** 2016-11-16

**Authors:** Shadi A. Ghassemi Jahani, Jon Karlsson, Helena Brisby, Aina J. Danielsson

**Affiliations:** 1 Department of Orthopedics, Frölunda Speciality Hospital, Västra Frölunda, Sweden; 2 Department of Orthopedics, Sahlgrenska University Hospital, Gothenburg, Sweden; 3 Department of Orthopedics, Institute of Clinical Sciences, Sahlgrenska Academy, University of Gothenburg, 413 45 Gothenburg, Sweden

**Keywords:** Thalidomide, Embryopathy, Long term, Outcome, Function, Quality of life

## Abstract

**Objectives:**

The aim of this study was to evaluate the effect of limb malformations on health-related quality of life (HRQL) and function of the extremities in middle-aged individuals with thalidomide embryopathy (TE). Between 1959 and 1962, approximately 150 children with multiple malformations were born in Sweden following the maternal intake of thalidomide during pregnancy, of whom 100 survived.

**Methods:**

Thirty-one individuals with TE underwent evaluations of musculoskeletal manifestations by clinical examination. Validated questionnaires were used for the assessment of general HRQL [the 36-Item Short Form Health Survey (SF-36) and the EuroQ Five Dimensions health questionnaire (EQ-5D)]. The function of the upper and lower extremities was evaluated using specific questionnaires (Disabilities of the Arm, Shoulder and Hand scale and Rheumatoid and Arthritis Outcome Score, respectively). The lower limbs were evaluated by computed tomography. The median age of the study group was 46 years, and 42% were females. Twenty-five individuals had malformations of the hand, but 27 had a grip function. Five individuals had severe lower limb malformations. Individuals with at least one extremity with major malformation(s) that affected function (*n* = 15) were compared with those without (*n* = 16).

**Results:**

The physical HRQL for the entire study group [mean 40.6, 95% confidence interval (CI) 35.4–45.8], as evaluated by the Physical Composite Score (PCS) of the SF-36, was significantly lower than the national norm value (population-based norm) of 50.0, and the physical HRQL of the subgroup with major limb malformations (15/31) was even lower (mean 34.6, 95% CI 25.9–43.4). The mental aspects of HRQL, based on SF-36 and EQ-5D scores, were not affected in the entire study group or in the subgroups.

**Conclusion:**

The physical quality of life was significantly lower in individuals with TE compared with the general national population, while the mental aspects were not affected.

**Level of evidence:**

IV.

## Introduction

Children with congenital or acquired conditions are forced to learn to manage their situation from the very beginning. Sometimes, despite treatment or surgical procedures, they enter adult life with functional deficiencies that will last their entire life. With aging, there is a risk of deterioration due to the development of degenerative changes. An individual with acceptable performance in early adulthood may have been on the border of his/her ability, but, with aging, this ability might be lost.

Over the years, thalidomide has been used to treat different medical conditions, such as leprosy and other painful conditions [1]. In the late 1950s and the beginning of the 1960s, thalidomide was offered as a treatment for insomnia and was frequently used by pregnant women due to its reputation of being harmless. However, when the number of children born with multiple malformations all over the world increased following the maternal intake of thalidomide during pregnancy [2, 3], it gradually became apparent that thalidomide was a highly teratogenic substance [4]. This led to the syndrome being referred to as thalidomide embryopathy (TE). Thalidomide was subsequently banned from the market for some years, but today it is once again being used for the treatment of leprosy [5, 6], multiple myeloma [7], Crohn’s disease [8], and other conditions. Despite our knowledge of the teratogenic effect of thalidomide, children still being born with TE [6]. Information on its teratogenic effects is still insufficient and action for the prevention of TE is not being taken.

Malformations induced by thalidomide include those affecting the ears, eyes, oral region, musculoskeletal system, and a variety of inner organs [9]. There is large individual variation in terms of the number and the severity of these malformations, and some children die before reaching adulthood. The malformations that do occur are not specific to TE, and all malformations can occur separately without any previous use of thalidomide. Information on the long-term outcome of individuals with major malformations, and especially on the long-term outcome of those with TE, is relatively scarce.

The children with TE born at the beginning of the 1960s have now reached middle age. Some studies of the long-term outcome related to a variety of the malformations have recently been published—on ocular problems [10], speech pathology [11], oral health including teeth [12] and the development of degenerative changes to the lower limbs and the cervical spine [13, 14]. Studies from Japan report that mental health had deteriorated and anxiety levels had increased in this population [15].

To date, there has been no published study on the effect of such extremity malformations on the health-related quality of life (HRQL). Knowledge relating to this specific long-term outcome would help to guide medical professionals on how to care for younger individuals with these types of extremity malformation, regardless of origin, and also help to predict the need for future assistance.

Instruments for the evaluation of HRQL are currently being used to describe different aspects of daily life. These instruments measure both the mental and the physical components of HRQL. However, to evaluate functions in a specific condition or disease, specific questionnaires are usually used. Malformations of the extremities, like those in TE, might affect daily living and thereby also HRQL. It is therefore of great interest to evaluate the function of the upper and the lower extremities using specific instruments.

The aim of this study was to evaluate HRQL in middle-aged individuals with TE using the following specific research questions: (1) Is the HRQL in middle-aged individuals with TE similar to that of the general population? (2) To what extent do limb malformations affect HRQL? (3) Does the function of the upper or the lower limbs, as measured by validated disease-specific questionnaires, correlate to general HRQL?

## Materials and methods

### Study design

The National Thalidomide Society was established during the 1960s when legal processes against the manufacturing company began. It includes all individuals who have been diagnosed with TE according to the described standards [16–18]. All individuals, apart from 24 individuals who had refused contact for any study purpose, were invited to participate in this study. Eighteen individuals refused to participate, 33 did not respond to the invitation, and 33 did accept the invitation to participate. Among these latter 22 individuals, two patients were not included in the study due to severe neurological disability (*n* = 1) and recent stroke just before the examination (*n* = 1). As a result, 31 individuals were enrolled in the study.

The study was multidisciplinary and, in addition to the orthopedic assessment, we included ophthalmology, otolaryngology, speech pathology, dentistry, and neuropsychiatry investigations. Individuals underwent a full clinical examination, a spiral computed tomography scan of the lower limbs, and an magnetic resonance imaging study of the cervical spine for the evaluation of existing malformations and the development of degenerative changes. Detailed descriptions of these findings have been published [13, 14].

### Study group–baseline data

The median age at follow-up was 46 (range (45.2–50.1)years, and 13 (42%) were female. Basic information relating to the malformations of the patients is given in Table 1. Twenty-seven individuals (87%) had malformations of the upper limbs and 25 (81%) had malformations of the hand(s). All but one individual had two existing hands with at least three fingers on each. Significant arm shortening was present in four individuals. Some grip function was found bilaterally in 27 individuals, but only 11 of these had a proper bilateral anatomical pincer grasp. Three individuals (10%) used orthotic devices of the upper limbs.

**Table 1:** Muskuloskeletal malformations found in 31 individuals with thalidomide embryopathy at follow-up of this study

Upper extremity	*n* (%)	Lower extremity	*n* (%)	Other malformations	*n* (%)
At any location of the upper extremity	27 (87)	PFFD in combination with other deformities^a^	5 (16)	Ear: hearing deficit	6 (19)
Shoulder	5 (16)	Hip deformity without a diagnosis of PFFD^b^	1 (3)	Facial nerve palsy	3 (10)
Elbow/forearm	11 (36)	Other malformations		Duane’s syndrome^c^	9 (29)
Hand	25 (81)			Incomitant gaze	10 (32)
Type of grip function				Tearing while eating	5 (16)
Anatomical pincer grip bilaterally	11 (36)			Other internal anomalies^d^	10 (32)
Any type of functional grip bilaterally	27 (87)				

All values in table represent occurrence in number of patients, regardless of whether it is uni- or bilateral

*PFFD* Proximal focal femoral deficiency

^a^See Table 2 for further description

^b^Dislocated hip, coxa vara, and femoral bowing before surgery with total hip replacement. The primary appearance is unknown

^c^Duane’s syndrome; a disorder in abduction of the eyes due to defect cranial nerve 6 (CN)

^d^Includes double vagina, aplasia of the uterus, kidney aplasia, choanal atresia and enamel hypoplasia

**Fig. 1:**
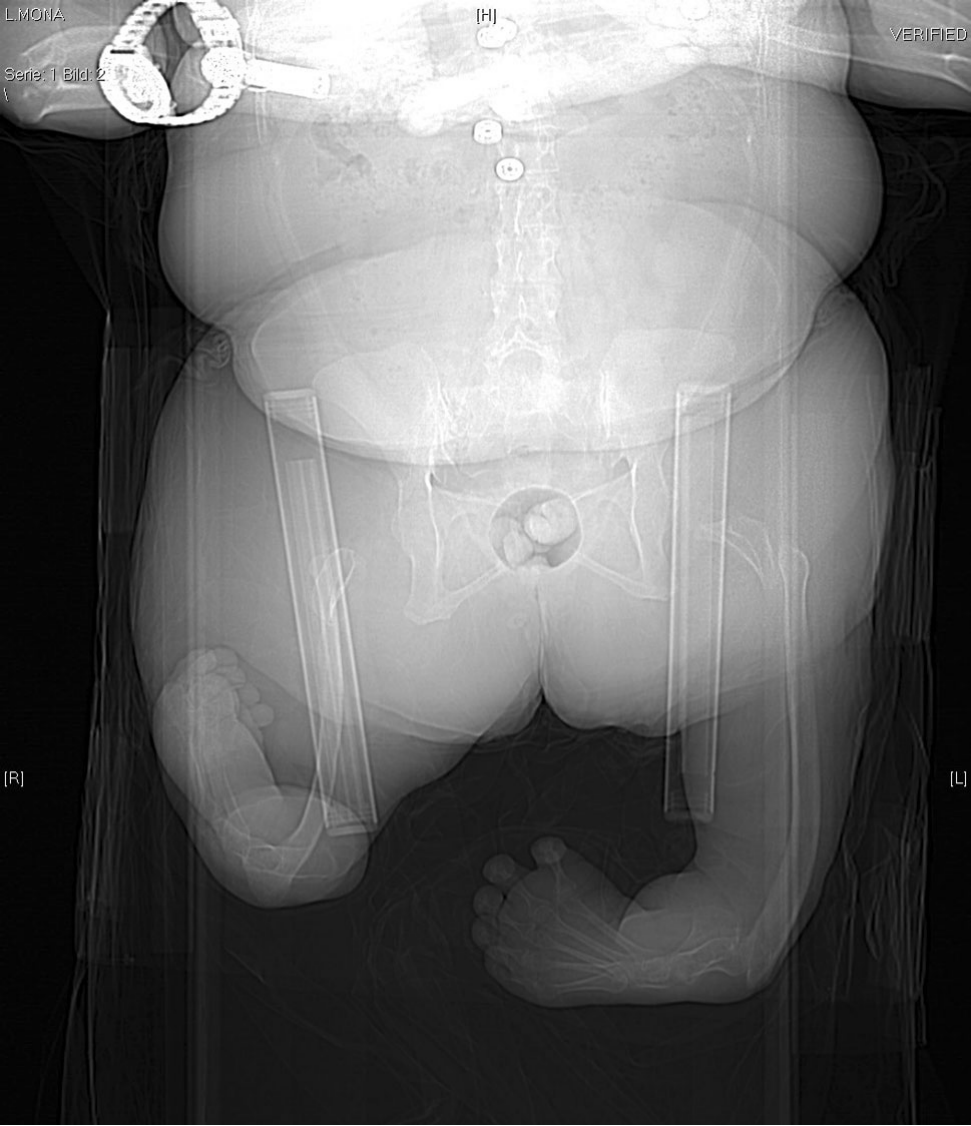
Computed tomography scan of one study participant with bilateral proximal focal femoral deficiency and fibular hypoplasia and tibial aplasia of the lower limbs

Few individuals had severe malformations of the lower limbs; five had a proximal focal femoral deficiency, PFFD [19, 20], which was bilateral in three of these individuals (Table 2; Fig. 1). Four of the five also had major lower leg malformations, such as tibia or fibular hypoplasia or aplasia. One lower limb had a normal anatomy, including the foot, but all of the lower limbs showed some type of foot deformity. No individual had undergone a Van Ness rotation plasty [21], reconstructive surgery, or lengthening procedures, but six other surgical procedures had been performed in this group. All individuals had a significant shortening of the lower limb(s), necessitating the use of prostheses and occasionally a wheelchair in four individuals (13% of the study group). All five patients with PFFD had several malformations of the upper extremities. The five individuals with PFFD were compared with the rest of the group (*n* = 26).

**Table 2:** Appearance of the lower limbs and previous surgery in the five individuals of the study group diagnosed with proximal focal femoral deficiency (Patient I–V)

Malformations of lower limbs/previous surgery	I		II		III		IV		V	
	Right	Left	Right	Left	Right	Left	Right	Left	Right	Left
Significant shortening	•	No	•	•	•	•	•	•	•	•
PFFD^a^	II/B	No	II/D	II/D	II/C	No	I/C	II/C	I/D	II/D
Tibia or fibular hypoplasia/aplasia	No	No	•	•	•	Absent	•	•	No	•
Major foot deformity?	Equinus, cavus	No	Valgus, abductus	Varus, supinatus	Only hindfoot	Absent	Equinus	Equinus, varus	Equinus	Equinus, varus
Previous surgery, all before age 18 years	No	No	No	No	Midfoot amputation	Amputation at knee level	No	Correction of equinus/varus deformity	Hip surgery^b^	Correction of equinus/varus deformity

^a^Description of the different grades of PFFD is according to Gillespie and Torode [20] and Aitken [19]. Gillespie and Torode [20]: Group A: the femur is up to 50% shorter than the normal femur and the hips and knees can be made functional. Group B: more severely shortened femora in which the foot on the affected side reaches above the mid-tibia on the normal side, often at the level of the normal knee. With the hips flexed, the femur is noted to be at or less than half the length of the contralateral femur. Group C: subtotal absence of the femur. Aitken [19]: Type A: the femoral head present in a normal acetabulum, short femur. Type B: the femoral head is present in the acetabulum, adequate, or moderately dysplastic acetabulum, femoral segment short. No osseous connection between head and shaft. Type C: femoral head absent or represented by ossicle, severely dysplastic acetabulum, femoral segment short. May be an osseous connection between the shaft and proximal ossicle. Type D: femoral head and acetabulum absent, short femoral segment, deformed. No relationship between different components of the femur and acetabulum at skeletal maturity

^b^The computed tomography scan showed a deformed proximal femur without a proper femoral head, located superior to the normal position of the acetabulum. No proper acetabulum or neo-acetabulum could, however, be seen. One staple left in the trochanteric region

For the purpose of this study, major malformations that were expected potentially to affect the function and/or quality of life (QoL) were used as a basis for evaluations. These major malformations included considerable shortening or deformity and/or severe functional loss. The latter was defined as a lack of proper gripping function in the upper extremity and/or as having difficulties with locomotion or positioning of the body to reach out due to shortening or deformity/ies of the lower extremities. Sixteen individuals had no major deformities of any of the four extremities, while 15 had major deformities affecting function in at least one extremity (one extremity in five individuals, two in nine individuals and four extremities in one individual).

### Questionnaires

In order to identify the impact of the condition and the individual disability, we used a number of generic and specific quality-of-life questionnaires. All the questionnaires have been validated previously and used for their specific purposes. A standardized procedure for questionnaire administration was followed [22]. All questionnaires were completed on the same occasion during a personal visit and prior to the clinical examination.

Sociodemographic issues were covered by questions relating to family situation, working conditions, and general health issues, including other diseases, pain, and medication.

#### Functional analysis

Function of the limbs was evaluated by specific questionnaires. The Disorder in Arm, Shoulder and Hand (DASH) scale was used to evaluate the upper extremity function [23, 24]. The results are presented as a disability/symptom score (the DASH score) for all 30 essential items and as two optional high performance scores, the Sport/Music and Work scales, each comprising four items. The score ranges from 1 (no difficulty performing an activity) to 5 (impossible to perform), and at least 27 of 30 questions must be answered. The results vary from 0 (best function, i.e., least disability) to 100 (greatest disability).

The Rheumatoid Arthritis Outcome Score (RAOS) [25] was used to evaluate the function of the lower extremities. Five separate patient-relevant dimensions, namely, pain, other symptoms (i.e., stiffness, swelling, and range of motion), activities of daily living (ADL), sport and recreational (Sport/Rec), and lower limb-related QoL are calculated after answering the self-administered 42-item questionnaire. Each item is graded as no, mild, moderate, severe, and extreme (scored as 0–4), and each of the five subscale scores are then calculated as the sum of the items included. Raw scores are transformed to a zero to 100 (worst to best) scale.

The DASH disability/symptoms score and the pain score of the RAOS were included in the correlation analyses.

#### General HRQL

General HRQL was evaluated using the 36-Item Short Form Health Survey (SF-36) questionnaire. The eight subscales and the Physical and Mental Composite Summary scores were calculated, resulting in 100 as the best possible score for the subscores and 50 as the norm for the composite scores. The SF-36 has been psychometrically well documented, as well as previously validated for use in our country [26]. The results are presented as the mean and 95% confidence interval (CI).

For the evaluation of the reported level of QoL as measured by the SF-36, the patients’ results were compared with the national norms, i.e., the general population. An individual *z*-score for each subscale of the individual participants in the study was calculated. In this analysis, the reference values, mean, and standard deviation (SD) for all subscales are first identified for the gender and 5-year age interval in the national SF-36 reference population that corresponds to the specific individual. Thereafter, the difference between the individual domain score value and the reference mean for that specific subscale is calculated. This difference is then divided by the reference SD. As a result, each subscale of the SF-36 for that particular individual receives its own* z*-score, which therefore reflects the difference for that specific individual compared with the normal population. These* z* scores were used for statistical calculations.

Further, the EuroQ Five Dimensions health questionnaire (EQ-5D) was also used to evaluate the general HRQL [27]. Validation for use in our country has been performed [28]. The results for this study group were compared with the results for the general population from the age group equivalent to the study group (age 40–49 years) [29].

### Statistical methods

The results relating to continuous variables as well as to changes in continuous variables were described with number (*n*), mean, SD, and range for each group. The results of the SF-36 were presented as the mean with the 95% confidence interval, while those of the EQ-5D were presented as the mean with the standard error, according to the most used standards for these questionnaires. The results for categorical variables, including dichotomous variables, were presented as a number and percentage.

The Mann–Whitney *U* test was used to compare continuous variables between two groups, and Fisher’s exact test was used to compare dichotomous variables between two groups. The Mantel–Haenszel χ^2^ test was used for comparisons between two groups involving ordered categorical variables, and the χ^2^ test was used to compare groups involving non-ordered categorical variables. Correlations were analyzed with Spearman’s rank correlation. All tests were two-tailed and conducted at the 5% significance level. Wilcoxon signed-rank test was used when comparing two related samples.

Statistical analyses were performed using version 9 of the SAS System for Windows (SAS Institute, Cary, NC) and version 19 of the Statistical Package for the Social Sciences (SPSS; IBM Corp., Armonk, NY).

## Results

### Sociodemographic data

Sociodemographic conditions are presented in Table 3. Twenty-four individuals (77%) were working at the time of the study, of whom 15 (15/31, 60%) worked full time. Despite major limb deformities, only four of 15 (27%) individuals were sick listed or retired. Only one individual, with major limb malformations, reported a living situation with heavy stress. Ten individuals reported the daily use of analgesics. The group with major limb deformity/ies did not differ from those without any major limb malformations for any of the variables listed in Table 3.

**Table 3:** Sociodemographic variables reported by 31 individuals with thalidomide embryopathy

Sociodemographic variables	All patients (*n* = 31)	Occurrence of major malformations in any of the limbs		
		No (*n* = 16)	Yes (*n* = 15)	*p* value
Family situation				
Marital status				
Never married	5 (16)	2 (13)	3 (20)	0.55 ns
Married/cohabitant	25 (81)	13 (81)	12 (80)	
Divorced/widowed	1 (3)	1 (6)	0	
No of individuals without children	9 (29)	5 (31)	4 (27)	0.89 ns
Education (highest achieved level)				
<7 years of school attendance	0	0	0	0.69 ns
High school (completed or not)	4 (13)	3 (19)	1 (7)	
Vocational school	3 (10)	1 (6)	2 (13)	
College (completed or not)	15 (48)	8 (50)	7 (47)	
Graduate school	9 (29)	4 (25)	5 (33)	
Working life				
Employment				
Currently working	24 (77)	13 (81)	11 (73)	0.41 ns
Housewife	1 (3)	1 (6)	0	
Sick listed or retired	6 (19)	2 (13)	4 (27)	
Working time				
Working full time (of those working)	15 (62)	9 (69)	6 (55)	0.32 ns
Stress				
No stress	7 (23)	3 (19)	4 (27)	0.47 ns
Minor stress	23 (74)	13 (81)	10 (67)	
Heavy stress	1 (3)	0	1 (7)	

Values in table are given as the number of patients with the percentage of the group under consideration given in parenthesis

*ns* Not significant

### Function of the upper and lower limbs, i.e., disease-specific HRQL

#### Upper extremities

The DASH disability/symptoms score, which evaluates the function of the upper extremities, was 20.5 (SD 15.6) for the entire study group. Significantly reduced upper extremity function was noted in the group *with* major limb deformity/ies (mean 25.3, SD 17.3) compared with those *without* any major limb deformities (mean 14.3, SD 12.1; *p* = 0.015). Likewise, the group *with* PFFD had a significantly reduced upper extremity function (mean 34.3, SD 6.3) compared with those *without* PFFD (mean 17.9, SD 15.5; *p* = 0.0040). The more detailed scores for work and for sports/music did not reveal any differences between the subgroups.

#### Lower extremities

**Fig. 2:**
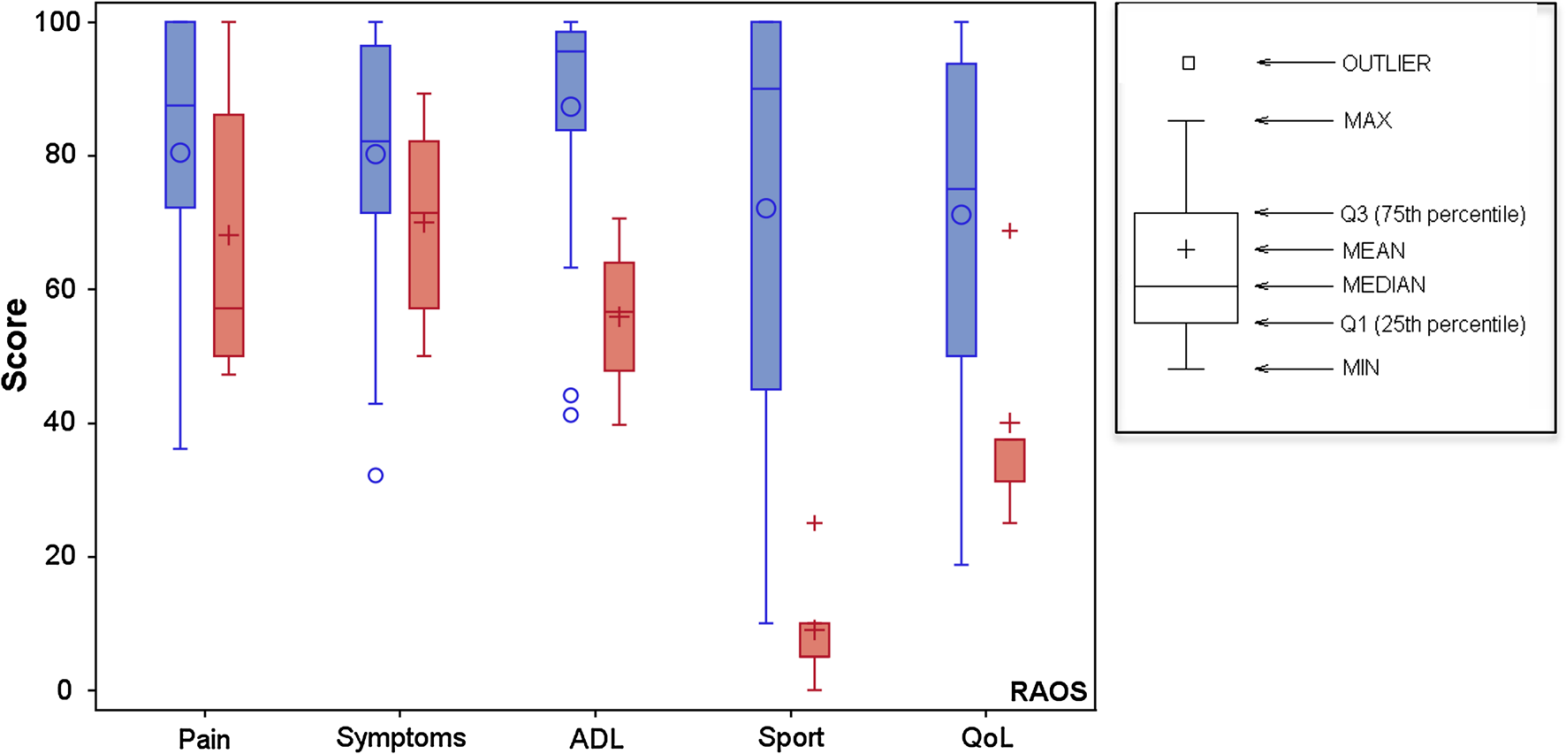
Results for the five subscales/dimensions of the Rheumatoid Arthritis Outcome Score (RAOS) in individuals with (*n* = 5, *red*) or without proximal focal femoral deficiency (*n* = 26, *blue*). RAOS subscales are: pain, other symptoms (i.e., stiffness, swelling, and range of motion;* Symptoms*), activities of daily living (*ADL*), sport and recreational (*Sport*), and lower limb-related quality of life (*QoL*)

The function of the lower limbs, as measured by the RAOS is presented in Table 4. The RAOS was not affected by the occurrence of major limb deformity/ies, but the group with PFFD had significantly reduced function based on the scores for ADL, sports and recreation (Sport/Rec), and lower limb-related QoL compared with those without PFFD (Fig. 2).

**Table 4:** Function of the lower extremity as measured by the Rheumatoid Arthritis Outcome Score in individuals with thalidomide embryopathy

Patient-relevant dimensions of RAOS	Individuals with TE^a^
Pain	78.5 (20.6) [36–100]
Symptoms	78.6 (17.9) [32–100]
Activities of daily life	83.1 (19.7) [40–100]
Sports and recreation	61.9 (36.4) [0–100]
Quality of life	66.1 (26.2) [19–100]

Values are presented as the mean score with the standard deviation in parenthesis and the range in square brackets. 100 is the best possible score.

*RAOS* Rheumatoid Arthritis Outcome Score,* TE* thalidomide embryopathy

^a^Data were available for 31 individuals in each dimension, with the exception of Activities of daily life for which data are available for 30 individuals

### QoL as determined using generic questionnaires

#### Physical and mental composite scores of SF-36

**Fig. 3:**
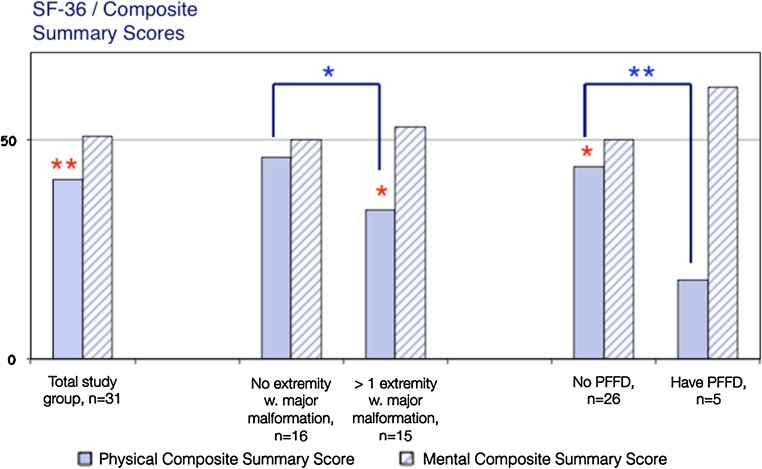
Physical Composite Scores of the 36-Item Short Form Health Survey (*SF-36*) in the entire study group and analyses of the subgroups. Summary scores of 50 represent a level regarded as normal (national norm). Comparisons in relation to national norms (*z*-scores): *p<0.05, **p < 0.01 (*red color*, located just above the respective *bar*). Comparisons between groups: *p<0.05, **p < 0.01 (*blue color*, located at the *top* of the figure).* PFFD* Proximal focal femoral deficiency

The physical composite summary score for the entire group (mean 40.6, 95% CI 35.4–45.8) was significantly reduced in relation to the national norm (population-based control group) (Fig. 3). Individuals with limb/limbs with major deformity/ies had a significantly lower physical HRQL as measured by the Physical Composite Score (PCS) of the SF-36 compared with those without any extremities with major deformities (mean 34.6 vs. 45.8, respectively; *p* = 0.040). The group with PFFD also reported a significant reduction in the PCS compared with the rest of the group (mean 18.3, vs. 44.0, respectively; *p* = 0.00031).

The mean Mental Composite Score was 51.5 (95% CI 47.1–56.0) for the entire study group and no differences were noted between the subgroups. Also, no differences were found in the* z*-score analyses in relation to the control group for the entire study group or the subgroups.

#### SF-36 subscores

**Fig. 4:**
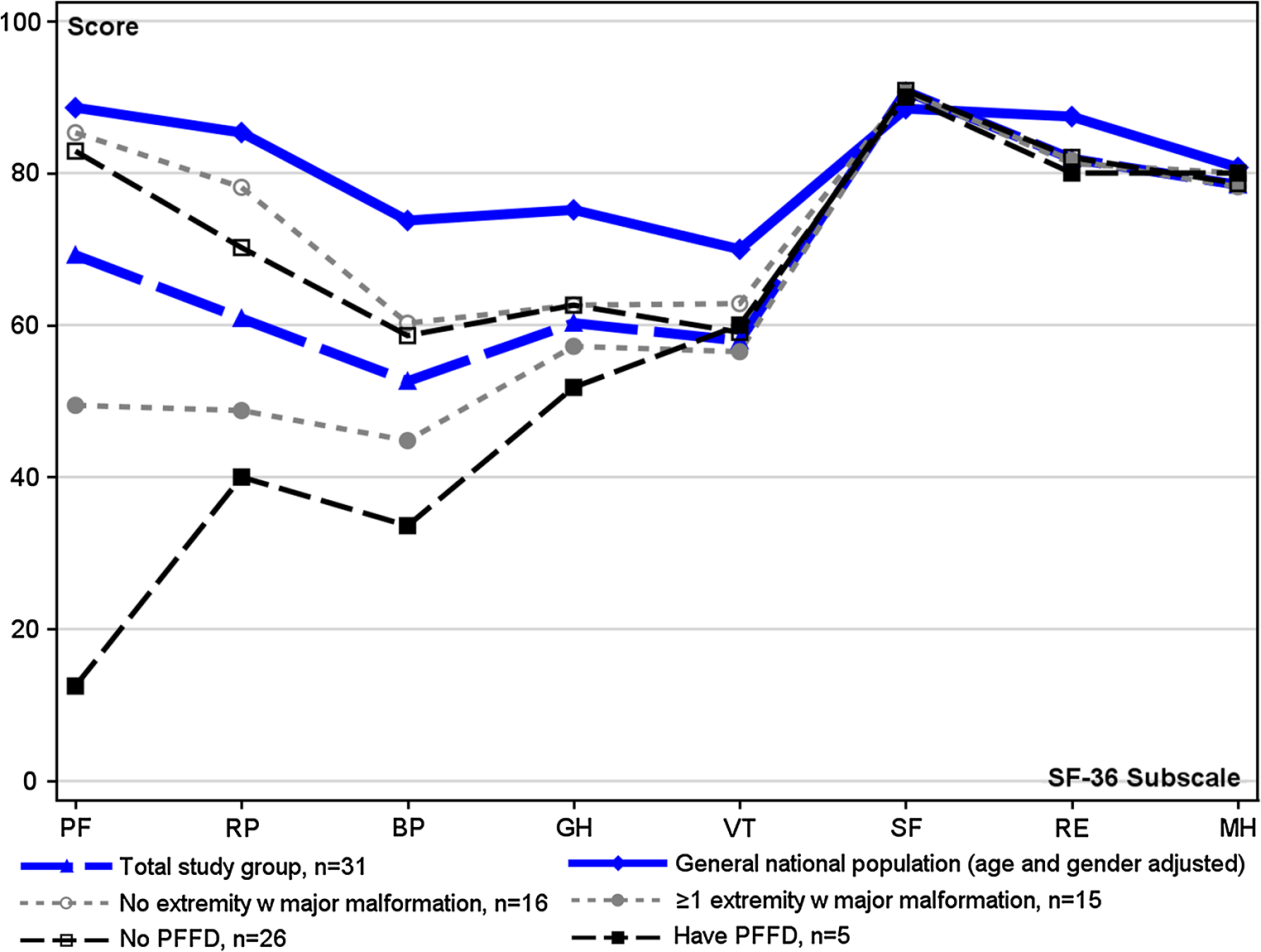
Subscores of the SF-36 for the total study group and for subgroups in terms of the occurrence of major malformations of the extremities or of PFFD. The results for the general national reference population adjusted for age and gender are also presented for reference

The outcome as HRQL as measured by the SF-36 is shown in Fig. 4. The entire study group reported low levels for physical QoL, with scores for the Physical (mean 65.3, 95% CI 51.4–79.3), Bodily Pain (mean 54.6, 95% CI 44.6–64.5), and General Health (mean 60.9, 95% CI 53.0–68.7) dimensions all significantly lower than those of the population-based control group. The mean Vitality dimension score was 59.2 (95% CI 51.4–67.0) for the total study group, which was also significantly lower than that of the population-based control group.

Individuals with and without major limb deformity/ies did not differ significantly from one another for any of the subscores. The* z*-score analysis revealed that the physical subscales and Vitality score of the group with major deformities were significantly different those of the general population. The mental subscales did not differ significantly between the subgroups or in relation to the healthy population.

The individuals *with* PFFD (*n* = 5) had a significantly poorer physical QoL than those* without *PFFD (*n* = 26), with the former having a lower mean Physical Functioning score [12.5 (95% CI −6.4 to 31.4) vs. 82.9 (95% CI 75.1–90.7)] and a lower mean score for Bodily Pain [33.6 (95% CI 18.0–49.2 vs. 58.6 (95% CI 47.5–69.7); *p* = 0.044]. The group *without PFFD* had significantly lower* z*-scores for Bodily Pain, General Health, and Vitality than did the general population. In the group *with* PFFD, the mean* z*-score for Physical Functioning was −4.2, which is fourfold lower than the standard deviation for the general population, but this difference was not statistically significant. For the mental subscales, no significant differences were noted between the two subgroups or when the subgroups were compared with the general population.

#### EQ-5D instrument

Table 5 shows the results of the EQ-5D analyses. The five dimensions of the EQ-5D were not affected by the presence of extremities with major deformities. The EQ-5D index was significantly lower in the group of individuals with PFFD than in those without (mean 0.35 vs. 0.66; *p* = 0.035). Moreover, for the Mobility and the Usual Activities subscales, a similar difference was observed.

**Table 5:** Health-related quality of life as measured by the EuroQ Five Dimensions health questionnaire in patients with thalidomide embryopathy

HRQL dimensions	Entire study group (*n* = 31)	Occurrence of PFFD			National population^a^
		No (*n* = 26)	Yes (*n* = 5)	*p* value	
Health profile					
Mobility^b^				0.018	
I have no problems walking about	24 (80.0%)	23 (88.5%)	1 (25.0%)		94%
I have some problems walking about	6 (20.0%)	3 (11.5%)	3 (75.0%)		6%
I am confined to bed	0 (0.0%)	0 (0.0%)	0 (0.0%)		
Self-care				1.00 ns	
I have no problems with self-care	28 (90.3%)	24 (92.3%)	4 (80.0%)		98.5%
I have some problems washing or dressing myself	2 (6.5%)	1 (3.8%)	1 (20.0%)		1.5%
I am unable to wash or dress myself	1 (3.2%)	1 (3.8%)	0 (0.0%)		
Usual activities				0.0098	
I have no problems performing my usual activities	23 (74.2%)	22 (84.6%)	1 (20.0%)		91.5%
I have some problems performing my usual activities	8 (25.8%)	4 (15.4%)	4 (80.0%)		8.2%
I am unable to perform my usual activities	0 (0.0%)	0 (0.0%)	0 (0.0%)		
Pain/discomfort				0.14 ns	
I have no pain/discomfort	5 (16.1%)	5 (19.2%)	0 (0.0%)		61.1%
I have moderate pain/discomfort	17 (54.8%)	15 (57.7%)	2 (40.0%)		38.9%
I have extreme pain/discomfort	9 (29.0%)	6 (23.1%)	3 (60.0%)		
Anxiety/depression				0.29 ns	
I am not anxious or depressed	22 (71.0%)	17 (65.4%)	5 (100.0%)		73.9%
I am moderately anxious or depressed	9 (29.0%)	9 (34.6%)	0 (0.0%)		26.1%
I am extremely anxious or depressed	0 (0.0%)	0 (0.0%)	0 (0.0%)		
EQ-5D Index	0.61 (0.12)	0.66 (0.12)	0.31 (0.41)	0.035	
EQ VAS rating	69 (3.0)	69.8 (7.1)	65.4 (3.4)	0.61 ns	

Values in table are presented as a number of patients with the percentage for that group in parenthesis or as the mean values with the standard error in parenthesis, as appropriate

*EQ-5D* EuroQ Five Dimensions health questionnaire,* VAS* visual analog scale

^a^ *n* = 30

^b^According to Burstrom et al. [29]

### Correlations between limb function and HRQL

The results from the correlation analyses are shown in Table 6. The physical QoL (as expressed by the SF-36 physical composite summary score) was found to correlate with the upper extremity function, as expressed by the DASH disability/symptoms score (*r*_s_ = −0.73, *p* < 0.0001), with the RAOS pain score for the lower extremities (*r*_s_ = 0.53, *p* = 0.0028) and with the number of extremities (*r*_s_ = −0.39, *p* = 0.035).

**Table 6:** Correlation analyses between health-related quality of life and functional scores in patients with thalidomide embryopathy according to Spearman’s rank correlation test

Generic questionnaires on HRQL	DASH ground score	RAOS pain	Number of extremities with major malformations (0–4)
SF-36			
Physical composite summary score	−0.72 (*p* < 0.0001)	0.53 (*p* = 0.0028)	−0.3 ( *p* = 0.035)
Mental composite summary score	0.17 ns (*p* = 0.36)	0.056 ns (*p* = 0.77)	0.085 ns (*p* = 0.66)
EQ-5D			
Aggregated score	−0.74 (*p* < 0.0001)	0.61 (*p* = 0.0003)	−0.18 ns (*p* = 0.33)
Health state today	0.15 ns (*p* = 0.41)	0.075 ns (*p* = 0.69)	−0.087 ns (*p* = 0.64)

*SF-36* 36-Item Short Form Health Survey

The aggregated score of the EQ-5D also correlated strongly with both the DASH Disability/Symptoms score and the RAOS Pain score.

## Discussion

To our knowledge, this study is the first long-term follow-up of HRQL in relation to limb deformities in individuals with TE. The main finding of our study is that the physical aspects of HRQL in many of these individuals were significantly lower than those of the national reference population, but that the mental aspects of HRQL were not significantly affected. The physical aspects of HRQL were found to correlate with the severity of the malformations. None of the individuals participating in this study had undergone the type of reconstructive surgery of the lower limbs which corrected for differences in limb length or relocated/stabilized the hip, knee, or ankle joint(s); consequently, the study group can be regarded as “untreated”. The results can therefore be seen as the natural history of these individuals and be used for a comparison of the outcome of current treatment strategies for lower limb malformations.

One important aspect of HRQL is whether differences occur in the study population in relation to the general population and to what extent. We used the SF-36 as the primary instrument to evaluate general HRQL as we had the opportunity to compare the study group and the national reference population using calculated* z*-scores. Compared to the national reference population, both the entire group and the subgroup with major limb deformities (according to either the number of limbs affected or the existence of severe lower limb malformations) had significantly reduced scores for the physical aspects of QoL. The subgroup with the lowest score for the physical aspects was the PFFD group, with a Physical Functioning score of only 12.5 compared with 88.6 in the reference population; however, this difference was *not* found to be significantly lower, despite being at a level of 4.3 SD below the reference population. The reason why such a large difference was not found to be significant can be explained by the fact that a comparison with a subgroup of only five individuals does not have the statistical potential to reveal these differences, even though they might truly exist. It is noteworthy that all of the differences observed between the TE group and the national reference group were above what has been considered to be a threshold for a clinically meaningful difference (6–8 points) [30].

The results of the EQ-5D from the general national population, presented as the percentage of individuals who reported moderate or severe problems, were used for comparison [29]. All dimensions involving physical activities revealed a lower level of HRQL, while the mental aspect (anxiety/depression) was unaffected, similar to the results found for the SF-36.

Two previous studies have reported results similar to ours for HRQL in individuals with TE. In 2002, Nippert et al. presented a report stating that 166 women with TE, aged 38 years, had significantly reduced physical QoL, as measured by the World Health Organization QOL-BREF instrument, but no reduction in psychological well-being [31]. Bent et al. reported that 41 individuals with TE, aged 40 years, had a physical function, as measured by the subscores of the SF-36, lower than their normative scores for the age group and that this was especially evident in individuals with a higher degree of perceived disability [32].

Interestingly, the mental aspects of QoL were not affected in the TE group participating in our study. This result may indicate that despite significant physical limitations in some cases, an acceptable level of mental well-being had been achieved, i.e., at a level similar to that of the rest of the national population. One possible explanation for this result may be that children who are born with congenital malformations do not know anything but the situation they have and have therefore adapted to it. Another contributory factor might be the support which society gives to TE individuals. When it was ultimately concluded that the malformations were caused by thalidomide, legal actions started against the distributing medical company and the National Thalidomide Society was set up. This resulted in the affected individuals not only receiving financial compensation, but also the attention and support of society in large. Since then, the Society has taken care of the interests of the group at all levels. In addition, it is possible that only individuals who had an acceptable mental HRQL may have chosen to participate in the study, which might have skewed the results towards a better outcome. A recent Japanese study [15] reported on 22 patients with TE and reported that 59% were assessed as having some kind of mental health problems. As this study focused solely on psychological and mental health problems, it used methods that were more specifically directed towards mental well-being and therefore more sensitive to the mental state than the relatively rough methods used in our study, which covers both mental and physical aspects. However neither the Japanese study nor our study reflects an entire national population with TE and in order to evaluate the true incidence of mental health problems, further studies will be needed. However, even if the present study does not reflect the true spectrum of mental well-being in the total TE population, it is still possible to use the results to evaluate the effects of limb malformations on mental well-being. Our results clearly show that despite a situation with reduced physical ability due to multiple malformations, it is possible to acquire a level of mental HRQL that is similar to that of the general population.

Other important aspects of QoL are the sociodemographic circumstances in which people live. The family situation and working conditions of our study group did not differ from those of general situation in Sweden. The study by Nippert et al. [31] reported rates of marriage/childlessness in the TE population as 42/63% and, in 2007, Bent [32] reported respective values of 70/46%, compared with our study results of 81/29%. More specifically, for working conditions, Nippert et al. [31] reported that 60% of the TE population were in full employment and 27% had retired, compared with our results of 48 and 19%, respectively. In terms of the reported stress levels, comparisons were more difficult, as this requires a more detailed discussion. However, the most important finding is that the occurrence of major deformities did not appear to impose any increase in stress on these individuals.

One limitation of our study is that other malformations or problems associated with TE, except for the limb deformities, might also affect HRQL. We therefore searched the results from the other parts of this multidisciplinary study in order to identify these factors. Duane’s syndrome was presented in 11 of 29 of the individuals examined in our study group, but they were found to be relatively mild and would therefore not affect the HRQL [10]. We therefore regarded the possible effect of such malformations on the level of QoL to be minimal and refrained from including this aspect in the analyses.

This study does not include the entire national population of individuals with TE. We do not know the incidence of malformations among those individuals with TE who did not participate, and this information has never been collected. The individuals who participated in our study do, however, present the full spectrum of malformations, and we were therefore able to analyze the effect of limb malformations on function and HRQL. As studies related to limb malformations and their consequences for the individual in the long term have not been published, we felt it was extremely important to present these findings. The results for the group of five individuals with PFFD, who were virtually untreated compared with current strategies involving extensive reconstructive surgery, can be regarded as natural history.

Taken together, the physical limitations significantly affected the physical HRQL in many individuals. No individual in our group of individuals with TE had received any reconstructive surgery of the lower extremities. Despite these physical limitations, most individuals actively participated in society, and their mental HRQL was at the same level as that of the general population. One possible explanation for this result is the major support received by these individuals from society, which includes financial compensation, obtained after the initial legal processes, and/or continued societal compensation when needed, as well as physical and psychological support, given by the families, environment and the national association. Modernized treatment strategies for orthopedic lower limb manifestations will help improve physical function in the future. Ackerman et al. reported on 12 individuals with PFFD only who were followed for a mean of 21 years after surgery with Van Nes rotation plasty performed at a mean age of 6.5 years. These authors reported physical function on the SF-36 that was similar to that of a collected age- and gender-matched control group, despite significant physical limitations among these 12 individuals [21]. Thus, with the improved treatment strategies for PFFD and tibia/fibula hypoplasia that are now available, which result in improved stability of the joints, reductions in limb length inequality, and less need for orthotic devices, physical function can be expected to improve substantially. This will hopefully further improve the physical long-term outcome for children who are born today with severe limb malformations or with TE.

Preventing the birth of children with TE is therefore crucial. Thalidomide is currently being used, seemingly with inadequate information on the well-known teratogenic properties, as children with TE are still being born today. Those responsible for treatment with thalidomide today must actively reduce the number of affected children, and if a child with TE is born despite these efforts, modern and continuous care and treatment must be ensured in order to achieve an acceptable level of QoL, both health-related as well as socioeconomic, throughout his/her lifetime. If these measures cannot be ensured, the continued use of thalidomide is truly unethical.

## Conclusions

The conclusions of the present follow-up at early middle age in this study group of individuals with TE are as follows.

1. The physical HRQL was significantly negatively affected in many individuals and correlated well with the existence of major limb deformities or with reduced function of the upper and lower extremities.
2. Despite overall affected HRQL, the mental aspects of QoL were not affected.
3. The majority of the middle-aged TE individuals were working, had a family, and did not experience large-scale stress.
